# Health Tract, No. 128

**Published:** 1865

**Authors:** 


### COFFEE POISONS.

If it be true that there are men so lost to all moral principle as to deliberately put strychnine and other poisonous drugs into liquid compounds, and then sell them for Bourbon whisky or French brandy, there are others who will adulterate coffee for the sake of gain, and sell it as a pure article. There are two very certain methods of avoiding imposition: either drink no coffee at all, or purchase the berry and burn and grind it yourself.

It is claimed that several families have been poisoned in Brooklyn by drinking what was sold for pure rye-coffee. Ergot of rye is certainly one of the most deadly poisons; and the city grocer may have been imposed upon by some careless farmer, who did not clean his grain properly. Those who are so lazy or thriftless as to purchase ground coffee to save themselves the trouble of preparing it at home, deserve to be poisoned — a little; but as it may be necessary sometimes to do so in an emergency, it is well to know that if ground coffee is pure, it very slowly discolors cold water, and is also slow to soften; but most adulterations blacken the water at once, and become soft besides. Of thirty-four samples of city-sold coffee of all kinds, thirty-one were found to be more or less adulterated.

"Chicory," or *succory*, is a garden endive, and is extensively used as coffee by the poorer classes; costing, in its parched and ground state, only fifteen cents a pound. It is simply the root of an herbaceous plant sliced, dried, parched and ground; it is one of the "drugs" of the apothecary, and is spoken of, in medical dispensatories as a "tonic;" as a "deobstruent;" as "acting on the liver;" it is said by some to impair digestion; to cause dyspepsia and bring on headaches, etc. The safer plan for all who wish to economize, and think they must have some kind of coffee for breakfast, is to use burnt bread-crust, or the common carrot prepared like chicory.

Many think they can not do without something to drink at regular meals; but this is a mere habit; if it must be done, it should be something quite warm, almost hot, because it is known by actual ocular demonstration, that cold water or any other cold liquid introduced into the stomach at meals, as instantly arrests the process of digestion, as water extinguishes a live coal; cold milk at meals has the additional disadvantage, if used freely, of engendering constipation, biliousness and the long list of minor symptoms which inevitably follow these conditions. But large draughts of even warm drinks at regular meal-times are very pernicious; as they not only cause "oppression," but by largely diluting the fluids which nature has prepared for converting the food into a nutrient material, render them less efficient, impose additional labor on the stomach and prematurely exhaust its powers. No one should exceed half a pint of liquid at any meal; invalids and the sedentary should use habitually still less.

